# The impact of yoga on components of energy balance in adults with overweight or obesity: A systematic review

**DOI:** 10.1002/osp4.552

**Published:** 2021-08-19

**Authors:** Ann E. Caldwell, Sarah A. Purcell, Bethany Gray, Hailey Smieja, Victoria A. Catenacci

**Affiliations:** ^1^ Division of Endocrinology, Metabolism, and Diabetes School of Medicine University of Colorado – Anschutz Medical Campus Aurora Colorado USA; ^2^ Anschutz Health and Wellness Center School of Medicine University of Colorado – Anschutz Medical Campus Aurora Colorado USA

**Keywords:** diet, exercise, energy metabolism, yogic

## Abstract

**Background:**

Yoga may reduce body weight in individuals with overweight or obesity, but whether this occurs through decreased energy intake (EI) or increased energy expenditure (EE)/physical activity (PA) is unclear.

**Methods:**

A systematic search of PubMed, Web of Science, Embase, and PsychINFO was conducted from inception until April 26, 2021. Eligible studies included randomized controlled trials or single‐arm pre‐post studies with any type and duration of yoga intervention in adults with overweight or obesity. Studies with measures related to EI , EE, or PA were eligible. The review initially identified 1,373 articles.

**Results:**

Of the 10 included studies, one used indirect calorimeter measures of resting EE, while nine used self‐reported measures of EI and PA. Of the seven studies measuring parameters related to EI, only one found greater decreases in EI relative to the control group, although three other investigations reported trends toward improved dietary intake. Of the eight studies measuring PA, two reported greater increases in resting EE or PA in the yoga group relative to the control group. Two reported significant within‐group increases in PA from pre‐post intervention, and four studies reported a trend for increased PA with no p‐values reported.

**Conclusions:**

Limited evidence suggests yoga may reduce EI and increase PA in adults with overweight or obesity. Additional studies that investigate the effects of yoga interventions on energy balance parameters using objective techniques are warranted.

## 1 INTRODUCTION

Improving lifestyle modification strategies for weight loss is an urgent public health priority due to the continued increasing prevalence of overweight and obesity,^1^, ^2^ the resultant incidence of comorbidities,^3^, ^4^, ^5^ and substantial financial burden on health care systems worldwide.^6^ Current obesity treatment guidelines recommend lifestyle modification interventions that include reducing energy intake (EI), increasing physical activity (PA), and enhancing counseling for behavioral modifications by trained interventionists.^7^ This lifestyle modification intervention approach typically produces 5%–10% short‐term weight loss, which provides significant health benefits.^7^ However, long‐term success in maintaining weight loss is poor with about 50% of lost weight typically regained within 1 year.^8^, ^9^, ^10^, ^11^ For lasting weight loss maintenance, obesity treatment guidelines recommend continued participation in a long‐term (≥1 year) comprehensive weight loss maintenance program.^7^, ^12^ It is therefore imperative to evaluate practical and cost‐effective long‐term strategies to improve the effectiveness of lifestyle modification interventions.

Yoga is a form of complementary medicine, that is, quickly growing in popularity in the United States^13^ and may be an effective strategy to improve the efficacy and durability of long‐term weight loss outcomes through lifestyle modification interventions. A recent systematic review and meta‐analysis of randomized controlled trials (RCTs) demonstrated that yoga interventions led to significant reductions in body mass index (BMI) in the subset of five studies that specifically included adults with overweight, obesity, or metabolic syndrome.^14^ However, whether lower BMI occurred through reduced EI, increased TDEE, or both has not been systematically investigated. This represents a considerable knowledge gap because understanding the extent to which yoga interventions impact specific energy balance parameters can inform the design of more effective lifestyle modification interventions.

There are several mechanisms through which yoga may theoretically improve energy balance.^15^ For example, yoga may help reduce EI by heightening mindfulness and the mind‐body connection, improving mood affect, and reducing stress.^16^, ^17^ Among individuals without obesity, regular yoga practice is associated with better dietary quality and improved management of emotional eating.^18^ In a qualitative study, individuals who lost weight through yoga described mindset shifts away from weight loss and toward health, increased mindfulness and focus, and improved self‐esteem.^19^ Improvements in self‐esteem and reductions in stress may be particularly relevant for mitigating the negative effects of weight stigma on weight loss, healthy eating, and PA.^20^ In addition, specific yoga postures may help increase EE directly, and by reducing some of the barriers to adopting and sustaining PA more broadly, as yoga can lead to reduced back and joint pain^15^ and improvements in physical function, isometric strength, cardiorespiratory fitness, and balance.^17^, ^21^ These unique psychological and physical changes associated with yoga interventions may therefore provide a form of activity, that is, more reinforcing for some individuals than higher intensity resistance or aerobic activities,^22^ as well as support changes in EI and moderate/vigorous intensity PA known to be critical for weight management. These elements may then facilitate longer‐term maintenance of dietary and PA changes after the intervention.

While these results are encouraging, most of the evidence supporting the positive effects of yoga on energy balance are from cross‐sectional or retrospective studies in populations without obesity. Understanding how yoga affects specific aspects of energy balance in people with overweight or obesity can inform novel and potentially more effective strategies for sustained weight loss. However, to date there has not been a systematic evaluation of the literature examining the extent to which yoga interventions lead to reduced EI, increased EE, or a combination of both in people with excess body weight. As such, the objective of this systematic review was to evaluate the existing literature examining the effect of yoga interventions on EI and PA among adults with overweight or obesity.

## 2 METHODS

### 2.1 Search strategy

This review was planned, conducted, and reported in accordance with the Preferred Reporting Items for Systematic Reviews and Meta‐Analyses (PRISMA) recommendations.^23^ A systematic literature search was conducted and included articles from inception until 26 April 2021 using PubMed, Web of Science, Embase, and PsychINFO. The search strategy entailed three independent themes of key words and medical subject heading (MeSH) terminology related to: (1) “yoga,” (2) “EI,” “PA,” or “EE,” and (3) “overweight” or “obesity” (Appendix 1 in the supporting information File). Search terms in each theme were linked using “OR” as a Boolean function and each theme was combined using “AND” as a Boolean function. To maximize study inclusion, no search limits on language of publication or record type were applied. Results from each database were managed using EndNote (Version X9, Clarivate Analytics, Philadelphia, PA, USA). The study team registered the study protocol on the International Prospective Register of Systematic Reviews (PROSPERO) database (CRD42020179845).

### 2.2 Study selection

Records uncovered during the literature search were assessed to determine if they met the following inclusion criteria^24^:

#### 2.2.1 Participants

Adults (age ≥ 18) with overweight or obesity, defined by body composition or any anthropometric measurement.

#### 2.2.2 Intervention

Yoga interventions of any duration or frequency and consisting of any combination of *asanas*, *pranayama*, or meditation/mindfulness.

#### 2.2.3 Comparator/control

Sedentary, dietary, or exercise control conditions. Single‐arm interventions were also included.

#### 2.2.4 Outcomes

All outcome measures related to EI, PA, or EE were eligible for inclusion. Specific eligible outcomes include dietary intake, hunger, satiety, appetite, dietary inhibition, dietary restraint, eating behavior, PA measured by any method, activity EE, resting metabolic rate, non‐exercise PA, or sedentary behavior. Notably, this article intentionally refers to the term “dietary intake” when describing specific dietary components or general food consumption and “EI” when referring to measured EI specifically (i.e., caloric intake) and with regard to energy balance more broadly throughout this manuscript.

Eligible publications included RCTs, quasi‐experimental studies, and single arm pre‐post studies. Cross‐sectional observational studies, conference papers, abstracts, dissertations, reviews, and non‐English publications were excluded. Three authors (H.S., B.G., and S.A.P.) independently sorted titles and abstracts. Potentially eligible records underwent full‐text review by A.C. and S.A.P. Two authors (A.C. and S.A.P.) also assessed the bibliographies of full‐text articles to identify other potentially eligible studies. Disagreements were resolved by review and consensus by A.C. and S.A.P.

### 2.3 Data extraction

Two authors (A.C. and S.A.P) independently extracted relevant data. Authors were not blinded to information regarding authorship, institutions of origin, or journal of publication. Information extracted included: First author last name, publication year, methods (study design, enrollment dates, length of follow‐up, randomization details [if applicable], and statistical analyses), participants (description, region/country, inclusion and exclusion criteria, and demographic and anthropometric characteristics), intervention, comparator/control groups, outcomes related to EI or EE, other outcomes, results (number of participants screened, number excluded, attrition, and EI and EE outcomes), conclusions by the authors, and references to other potentially relevant publications. This review reported PA with or without the yoga sessions, according to what parameter was included within each eligible article. Where possible, the team extracted the means and corresponding standard deviation, standard error, or 95% confidence intervals (CI) for each pre‐post‐ outcome variable.

### 2.4 Risk of bias

The National Institutes of Health’s study quality assessment tools were used to determine risk of bias.^25^ The checklists are designed to help reviewers determine internal validity for before‐after studies with no control group and RCTs through 12 and 14 questions, respectively. Two authors (A.C. and S.A.P) independently evaluated each study using the appropriate checklist. Discrepancies were resolved through discussion between researchers. Results are presented for each question (“yes,” “no,” “cannot determine,” “not applicable,” or “not reported”) and as an agreed‐upon overall risk of bias (low, moderate, or high, corresponding to the tool’s terminology of poor, fair, and good study quality, respectively).

## 3 RESULTS

### 3.1 Description of studies

**FIGURE 1:**
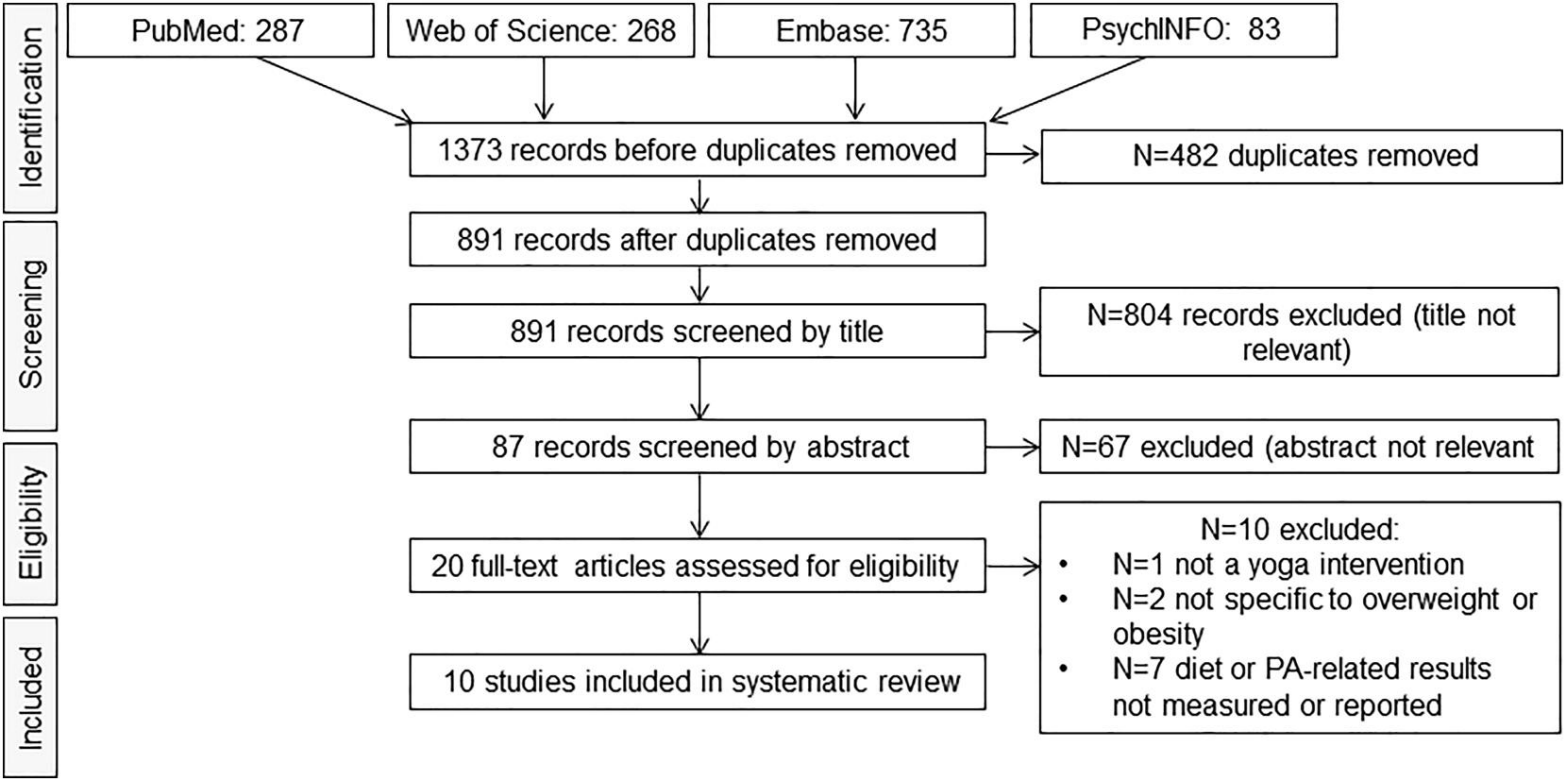
Flow chart of eligible and included publications. PA, Physical activity [Correction added on 28 August 2021, after first online publication: Figure 1 has been updated]

The literature search uncovered 1373 potentially eligible articles. After removing 482 duplicates and screening titles, abstracts, and full texts, 10 articles met inclusion criteria, Figure 1. These included eight RCTs^26^, ^27^, ^28^, ^29^, ^30^ and two pre‐post studies.^31^, ^32^ Of note, the article by Telles et al.^32^ included two study groups but was categorized as a pre‐post design due to the lack of randomization and comparison between groups. In addition, two RCTs recruited individuals with metabolic syndrome as opposed to overweight or obesity defined by BMI. These were included in this review because (1) high waist circumference is one of three criteria needed for metabolic syndrome and is also an indicator of central obesity and (2) first line of treatment for metabolic syndrome is changes in dietary intake and PA, similar to obesity treatment.

Table 1 outlines the details of the included studies. Interventions were conducted between 2007 and 2018 and ranged in length from 5 days to 1 year. Among the eight RCTs, 403 individuals were randomized to a yoga intervention, with 287 participants completing the intervention; 390 were randomized to control conditions, with 277 completing the studies. Among pre‐post studies, 66 individuals started the yoga interventions and 57 completed each yoga intervention, with 44 completing follow‐up measures.

**TABLE 1:** Descriptions of included studies and results for dietary intake and physical activity

First author, year	Study design	Intervention	Control	Population	Outcomes	Results	
						Dietary intake^a^	Physical activity
Braun et al.^31^	Pre‐post	5 days intensive workshop with Kripalu yoga methods (self‐acceptance, relaxation, *asana*, *pranayama*) and nutrition and lifestyle lectures. Included 12‐weeks follow‐up.	N/A	*N* = 37 adults age 32 to 65 with overweight or obesity (BMI > 25) in the USA	EI: Modified nutrition subscale (breakfast frequency, low‐fat, fruit, vegetable intake)	↑ in nutrition score after 5‐days program (2.71 ± 0.66, 3.25 ± 0.64, *p* = 0.001) ↑ in nutrition score after 12‐weeks (2.82 ± 0.56; 3.14 ± 0.47, *p* = 0.014), but not significant after Bonferroni correction	↑ in PA score after 5‐days program (2.19 ± 0.73; 2.61 ± 0.77, *p* < 0.001) ↔ in PA score after 12 weeks (2.20 ± 0.71, 3‐months: 2.31 ± 0.85, *p* = 0.529)
		*n* = 31 completed post‐program surveys, *n* = 18 completed 12‐weeks follow‐up surveys.			PA: Subscale, both from the health‐Promoting lifestyle Profile II		
Cohen et al.^26^	Randomized controlled trial	10 weeks of restorative yoga; 3‐hour introductory class, 90‐minute class 2×/week (weeks 1–5), 1×/week (weeks 6–10) in‐person	No intervention control	*N* = 26 underactive adults age 30 to 65 with overweight or obesity, meeting criteria for metabolic syndrome^b^ in the USA	Total EI, % EI from carbohydrate, fat, and protein via 2005 Block FFQ (macronutrient results not presented)	↔ in EI ∆ between groups (yoga: 233 ± 1462; control: 154 ± 546, *p* = 0.86); within‐group *p‐*values not reported	↔ ∆ between groups in hours/week (yoga: 2.0 ± 9.1; control: 1.6 ± 3.9, *p* = 0/89) or METs/week (yoga: 114 ± 1611; control: −47 ± 597, *p* = 0.75); no within‐group *p*‐values reported
		*n* = 14 randomized, *n* = 12 completed	*n* = 12 enrolled and completed		PA in hours/week and METs/week using the modified CHAMPS PA questionnaire		
Jakicic et al.^33^	Randomized controlled trial	24 weeks of either restorative or Vinyasa yoga; 5×/week, building from 20 to 60 minutes with one in‐person and four at home classes + EI restriction of 1200 to 1800 kcal/day + weekly behavioral weight loss lessons	Comparison group (not control) ‐ “Hatha Restorative” yoga	*N* = 50 adults with overweight or obesity in the USA	Total EI, % EI from carbohydrate, fat, and protein via diet history questionnaire (food frequency)	In both groups: ↓ in EI, kcal/day: Vinyasa: Baseline: 1471 (95% CI: 1084–1858), 6 months: 1054 (750–1359); restorative: 1503 (1076–1930), 1092 (788–1396); *p* = 0.005 time effect ↓ fat, % EI: Vinyasa: 36.3 (32.8–39.2), 29.6 (26.6–32.8); restorative: 36.0 (32.8–39.2), 31.9 (28.9–35.0); *p* = 0.0002 time effect ↑ carbohydrate, % EI: Vinyasa: 49.0 (44.9–53.1), 54.8 (50.4–59.2); restorative: 48.6 (44.5–53.8), 50.6 (46.4–54.8); *p* = 0.0284 time effect ↑ eating behavior Inventory: Vinyasa: 54.9 (51.2–58.6), 66.6 (62.7–70.5); restorative: 54.5 (50.6–58.3), 64.8 (61.0–68.6); *p* < 0.001 time effect	↑ PA in both groups and was greater in restorative yoga group: PA, kcal/week: Vinyasa: 596 (403–789), 831 (498–1164); restorative: 585 (387–783), 1189 (857–1521), *p* < 0.001 time effect; *p* = 0.0462 group × time PA, kcal/week, without stairs: Vinyasa: 322 (145–500), 489 (179–800); restorative: 292 (110–475), 877 (568–1186), *p* < 0.0001 time effect; *p* = 0.0178 group × time PA min/week, without stairs: Vinyasa: 57 (81–93), 102 (35–169); restorative: 52 (15–88), 192 (125–260), *p* < 0.0001 time effect; *p* = 0.0288 group × time
		In Vinyasa: *n* = 25 randomized, *n* = 23 completed	n = 25 randomized, n = 20 completed		Eating behavior Inventory		
					PA in kcal/week with or without flights of stairs and min/week without flights of stairs via validated questionnaire (not specified)		
Littman et al.^27^	Randomized controlled trial	24 weeks of Hatha‐based yoga for people with obesity; 5×/week, with 1 to 3, 75 minutes in‐person sessions; remaining sessions were 20 to 30 minutes at home	No intervention control	*N* = 63 post‐treatment stage 0–III breast cancer survivors age 21 to 75 with overweight or obesity (BMI ≥24 or ≥23 if of Asian descent) in the USA	PA in MET‐hours/week via self‐administered version of the Modifiable activity Questionnaire	N/A	↑ in MET‐hours/week of total PA in yoga group: pre: 15.1 ± 11.7, post: 19.2 ± 19.1; control: 12.4 ± 12.8, 12.1 ± 13.6; no *p*‐values reported ↑ in MET‐hours/week for non‐yoga PA: yoga: 15.0 ± 11.6, 16.9 ± 18.9; control: 12.4 ± 11.8; 12.0 13.6; no *p*‐values reported
		*n* = 32 randomized, *n* = 27 completed	*n* = 31 randomized, *n* = 27 completed				
Mama et al.^35^	Randomized controlled trial	8 weeks of culturally adapted movement‐based mind‐body intervention; 4×/week, with two 45 minutes in‐person sessions and two at‐home sessions (unspecified duration).	Waitlist control	*N =* 50 African American adults age 18 to 65 with overweight or obesity in the USA	Walking, moderate, vigorous, and total MET‐minutes/week from +MVPA in min/day via IPAQ‐long form	N/A	*Presented as change in parameters* ↑ in MET min/week: yoga: walking (2620), moderate (2371), vigorous (3153) total (8144) PA and MVPA in min/day (141); control: Walking (869), moderate (453), vigorous (57) PA and MVPA in min/day (17); no *p*‐values reported ↔ MVPA from accelerometer: yoga: −2 minutes/day; control: 0 minutes/day; no *p*‐values reported
		*n* = 26 randomized, *n* = 24 completed	*n* = 24 randomized, *n* = 16 completed		MVPA in min/day from accelerometry (waist‐worn Actigraph GT3X)		
Ruby et al.^28^	Randomized controlled trial	12 weeks consisting of 1 week of baseline testing and 11 weeks of yoga *asana* and relaxation: three, ≤1‐hour sessions/week; first 2 weeks in‐person, remaining 9 weeks were at‐home + Pilates	(1) Resistance training, three, ≤1‐hour sessions/week in conjunction with protein pacing diet *n* = 10 enrolled and completed (2) Protein pacing diet only	*N* = 27 women age 25 to 60 with overweight or obesity in the USA	Total EI, % EI from carbohydrate, fat, and protein, saturated fat, cholesterol, iron, glycemic index via 3 days food records	↔ in dietary parameters (change in values not presented)	N/A
		*n* = 8 randomized and completed	*n* = 9 enrolled, *n* = 7 completed				
Siu et al.^29^	Randomized controlled trial	1 year intervention consisting of three, 1‐hour instructor‐led Hatha‐based yoga sessions/week including *asana*, breathing and relaxation	No‐intervention control; subjects were contacted monthly to assess their health status	*N* = 283 adult males and females age 30 to 80 in China diagnosed with metabolic syndrome^b^	EI, carbohydrate, fat, protein, fiber, soluble fiber, sugar, and cholesterol via 3 days food records	↔ in dietary parameters: EI, kcal/day change: yoga: 64 ± 880; control: −126 ± 599, *p* *= 0.244* Carbohydrate, g/day: yoga: pre: 362 ± 148, post: 363 ± 177; control: 341 ± 148, 397 ± 426, *p* = 0.403 Fat, g/day: yoga: 74 ± 118, 50 ± 21; control: 61 ± 71, 61 ± 73, *p* = 0.834 Protein, g/day: yoga: 118 ± 62, 109 ± 45; control: 104 ± 45, 109 ± 76, *p* = 0.785	*Presented as change in parameters* ↑ PA in both groups; ↔ ∆ between groups: Total PA, MET‐minutes/week: yoga: 458 ± 2841; control: 358 ± 3598; no *p*‐values reported for time effect Sitting, min/week: yoga: −200 ± 1192; control: −348 ± 1560; no *p*‐values reported for time effect
		*n* = 146 randomized, *n* = 84 completed	*n* = 137 randomized, *n* = 98 completed		PA in MET‐minutes/week via IPAQ		
Telles et al.^32^	Pre‐post	12‐weeks intervention with three, 75‐minute in‐person sessions/week including *asana*, *pranayama*, guided relaxation, and meditation + lacto‐vegetarian diet plan of 1900 to 2000 kcal/day*n* = 29 enrolled in yoga, *n* = 26 completed	Nutrition advice group; received 1.45‐minutes nutrition lecture/week and same diet plan as yoga group*n* = 29 enrolled, *n* = 26 completed	*N* = 58 adult women age >20 with central obesity (waist circumference ≥ 80 cm and BMI ≥ 25)in India	EI, carbohydrate, fat, and protein (g/day) via 24‐hour diet recall; “eating behaviour” via Moorehead‐Ardelt quality of life Questionnaire^c^Estimated total energy expenditure via IPAQ^d^	↔ in dietary parameters between groups (no *p*‐values reported): EI, kcal/day: yoga: 1754 ± 424, 1591 ± 366; control: 1626 ± 395, 1716 ± 385 Carbohydrate, g/day: yoga: 217 ± 55, 249 ± 114; control: 194 ± 77, 222 ± 78 Fat, g/day: 39 ± 14, 41 ± 17; control: 48 ± 20, 47 ± 19 Protein, g/day yoga: pre: 59 ± 14, post: 51 ± 10; control: 54 ± 17, 56 ± 10 ↑ focus on eating behavior in yoga group: yoga: 0.2 ± 0.3, 0.3 ± 1.9; control: 0.3 ± 0.2, 0.3 ± 0.2	N/A
Yadav et al.^30^	Randomized controlled trial	12‐weeks intervention, *Patanjali ashtanga* based including *asana*, *pranayama*, relaxation, and meditation	Diet‐only group	*N* = 260 adult males and females, age 20 to 45, diagnosed with metabolic syndrome^e^ in India	EI; % EI from carbohydrate, fat, and protein; fiber (g/day) via 24‐hour diet recalls	*Presented as change in parameters* ↓ EI, kcal/day: yoga: 400 (95% CI: −506, −295), *p* < 0.001; control: −238 (−304, −171), *p* < 0.001; *p* = 0.010 group × time ↑ carbohydrate, % EI: yoga: 2.0 (0.9, 3.2), *p* < 0.001; control: 1.7 (2.8, 6.2), *p* = 0.003; *p* = 0.675 group × time ↓ fat, % EI: yoga: −2.9 (−4.0, −1.8), *p* = 0.001; control: −2.4 (−3.5, −1.3), *p* < 0.001; *p* = 0.499 group × time ↑ protein, % EI: yoga: 1.5 (1.1, 1.8), *p* < 0.001; control: 1.2 (0.8, 1.7), *p* < 0.001; *p* = 0.456 group × time	*Presented as change in PA*
		5×/week; first 2 weeks in‐person (∼2 hours), remaining 10 weeks at‐home + Tailored diet plan from a registered dietician	*n* = 130 randomized, *n* = 79 completed		PA in MET‐minutes/week via IPAQ‐short form		↑ PA, in MET min/week: yoga: 857 (189, 1658), *p* < 0.001; control: 693 (0, 1236), *P* < 0.001; *p* = 0.027 group × time
		*n* = 130 randomized, *n* = 89 completed					
Yazdanparast et al.^34^	Randomized controlled trial	8‐weeks intervention, with Hatha yoga (∼200 kcal energy expenditure) + 300 kcal/day EI restriction (yoga + diet)	Diet‐only intervention: 500 kcal/day EI restriction	*N =* 44 women with overweight or obesity (BMI ≥ 25 kg/m^2^) in Iran	RMR: Desktop indirect calorimeter (FitMate PRO)	N/A	*Presented as change in RMR*
		7×/week; 5×/week 60‐minute in‐person, 2×/week at‐home	*n* = 22 randomized; *n* = 18 completed				RMR, kcal/day: yoga: 95 ± 105; control: 0 ± 110, *p* = 0.010
		*n* = 22 randomized; *n* = 20 completed					

*Notes*: ∆: change; ↑: increase; ↓ decrease; ↔: no difference.

Abbreviations: BMI, body mass index; CHAMPS, Community Health Activities Model Program for Seniors; EI, energy intake; FFQ, food frequency questionnaire; in, kg/m^2^; IPAQ, International Physical Activity Questionnaire; MET, metabolic equivalency of tasks; PA, physical activity.

^a^ Energy intake and macronutrients reported for brevity (no micronutrients).

^b^ National Cholesterol Education Program criteria, defined as three or more of the following indicators: fasting blood sugar ≥100 mg/dL; systolic blood pressure ≥130 mmHg, diastolic blood pressure ≥85 mmHg, or use of antihypertensive medication; high density lipoprotein cholesterol (HDL‐C) ≤40 mg/dL for men or ≤50 mg/dL for women; triglycerides ≥150 mg/dL; and waist circumference ≥102 cm for men or ≥90 cm for women.

^c^ Telles et al.^32^ described food records as “weighed dietary records”; however, because no additional information on weighing methods or diet data analysis were provided, we assumed this was a 3‐days diet record without food weighing.

^d^ The IPAQ was administered, but MET‐minutes or MET‐hours per week were not reported and only used to calculate estimated total daily energy expenditure and thus results are not included.

^e^ Joint Interim Statement criteria, defined as three or more of the following indicators: fasting blood sugar ≥100 mg/dL, systolic blood pressure ≥130 mmHg, and/or diastolic blood pressure ≥85 mmHg, or use of antihypertensive medication; HDL‐C <40 mg/dL for men or <50 mg/dL for women; triglycerides ≥150 mg/dL; and waist circumference ≥90 cm for men or ≥80 cm for women (for Asians).

Yoga interventions varied widely in the styles and specific body‐, breath‐, and mind‐based practices included, as well on the level of detail provided to interpret the style of yoga or practices included in the intervention. All studies included body‐based yoga postures, or *asanas*, with three studies focusing exclusively on yoga postures.^28^, ^33^, ^34^ The intensity of the *asana* practices included in the interventions varied from being low intensity—described as restorative, therapeutic, or a means of stretching^26^, ^33^, ^35^—in three interventions, with six interventions providing descriptions indicating a more physically challenging *asana* practice.^28^, ^29^, ^30^, ^32^, ^33^, ^34^ Two studies specified using *pranayama* controlled breathing techniques,^30^, ^32^ which were only thoroughly described in the study by Telles et al.^32^ One study briefly mentioned breathing as part of the warm‐up, four studies described a focus on breath during postures^31^ or relaxation.^27^, ^29^, ^35^ Three studies specified including meditation techniques in line with classical Patanjali teachings,^30^, ^31^, ^32^ while one simply described meditation as part of relaxation,^27^ and another included meditations focused on Bible verses during relaxation.^35^

### 3.2 Energy intake outcomes

Seven studies measured some dimension of dietary intake before and after a yoga intervention.^26^, ^28^, ^29^, ^30^, ^31^, ^32^, ^33^ Of these, five studies combined yoga with dietary guidelines or advice,^28^, ^30^, ^31^, ^32^, ^33^ while two studies did not provide any instructions on diet and explicitly asked participants to *not* change their diet during the intervention.^26^, ^29^ All studies assessed dietary intake or nutrition via self‐reported measures, including a modified nutrition‐behavior questionnaire,^31^ the Eating Behavior Inventory,^33^ food frequency questionnaire,^26^ 24‐hour recall,^30^, ^32^ or 3‐days food records.^28^, ^29^ No studies used more rigorous measures of free‐living appetite or dietary intake (e.g., measures of hunger and satiety, doubly labeled water, etc.).

Of the five studies in which participants were given dietary advice, four reported changes in dietary intake and one found no change. In a large 12‐weeks RCT reported by Yadav et al.^30^ all participants (*n* = 260) received a personalized diet plan by a registered dietitian. In this study 130 individuals were randomized to also receive a yoga intervention 5 days/week in‐person and at‐home. Both groups had decreased total EI and % of EI from fat, increased % EI from protein and carbohydrates, and increased fiber (g/day). The yoga group had greater reductions in total EI. In a recent RCT by Jakicic et al.^33^ 50 adults with overweight or obesity were randomized to either a Vinyasa or restorative yoga for 24 weeks. Both groups received instructions on EI from 1200 to 1800 kcal/day. Both groups decreased EI and % of EI from fat and increased % of EI from carbohydrates; the Eating Behavior Questionnaire score also increased. In a small RCT (*n* = 26) among women with overweight or obesity by Ruby et al.^28^ participants in all intervention groups were given macronutrient‐balanced meal plans (50% carbohydrate, 15% protein, and 25% fat) designed to meet 100% of their estimated energy requirements. Following the 12‐weeks yoga intervention (3 days/week in‐person at‐home), there were no changes in dietary parameters attained from 3‐days food records.^28^ In a pre‐post pilot study by Telles et al.^32^ women with abdominal obesity (*n* = 29) received a 12‐weeks yoga intervention (3 days/week, in‐person) and a lacto‐vegetarian diet plan consisting of 1900 to 2000 kcal/day. EI appeared to decrease with concomitant decreases in protein and increases in carbohydrate and fat intake (in g/day) in the yoga group.^32^ However, as this was a pilot study, no *p*‐values were presented. In a single‐arm, 5‐days residential yoga study by Braun et al.^31^ participants attended yoga and lifestyle/nutrition classes centered around mindful eating that included cooking demos and meal planning (*n* = 39). A general “nutrition score” improved directly following the brief intervention and after 12 weeks; however, the 12‐weeks change was not significant after Bonferroni correction.^31^

The RCTs by Siu et al.^29^ and Cohen et al.^26^ explicitly asked participants to not change dietary intake. Siu et al.^29^ randomized 146 people to a 1‐year yoga intervention (3 days/week in‐person) or a no intervention control (*n* = 137). There were no significant differences self‐reported EI, macronutrients, sugar, or cholesterol attained by 3‐days food records between groups. Notably, self‐reported EI assessed by the Block Food Frequency Questionnaire (FFQ) appeared to increase in both yoga and wait‐list control groups in the pilot study by Cohen et al.^26^ However, no within‐group *p*‐values were presented and the differences in EI change between groups was not significant.

### 3.3 Physical activity and energy expenditure

Seven studies measured PA and one study measured resting metabolic rate before and after a yoga intervention. In six of these studies,^27^, ^30^, ^31^, ^33^, ^34^, ^35^ participants were provided a yoga intervention but not given instructions or advice to change habitual non‐yoga PA levels. Participants were provided a yoga intervention and explicitly instructed *not* to change habitual non‐yoga PA levels in two studies.^26^, ^29^ Six studies used self‐report measures of PA including the International Physical Activity Questionnaire,^30^, ^31^, ^32^ the Modifiable Activity Questionnaire,^27^ a modified Community Health Activities Model Program for Seniors (CHAMPS) Questionnaire,^26^ the PA subsection of the Health Promoting Lifestyle II questionnaire,^31^ and one questionnaire was not named.^33^ One study measured PA using accelerometers (in addition to the International Physical Activity Questionnaire)^35^ and another measured resting metabolic rate with a portable indirect calorimeter.^34^ No studies used objective measures of total EE (i.e., indirect calorimetry or doubly labeled water), or psychological factors predictive of PA (motivation or enjoyment). Of note, six of the seven studies reported PA outcomes in MET‐minutes or MET‐hours per week,^26^, ^27^, ^29^, ^30^, ^33^, ^35^ one included both total PA and non‐yoga PA,^27^ and Braun et al.^31^ reported a “PA score.” In the pre‐post study by Telles et al.^32^ MET‐minutes or MET‐hours per week from the IPAQ were not reported, but instead used to calculate estimated total daily EE with estimated basal metabolic rate using the Harris Benedict equation.^36^ Given that weight is included in this predictive equation (and weight changed significantly from baseline to post‐intervention in the study) and the study lacked essential details regarding PA calculations in the estimation of EE, the changes in estimated daily EE were not included in the interpretation of PA results.

In the six studies in which participants were not given instructions to keep habitual levels of PA the same, all six reported initial increases in PA or resting metabolic rate following the intervention, although not all were statistically significant. In the large RCT by Yadav et al.^30^ both the yoga and control groups significantly increased self‐reported MET‐minutes of PA per week; however, there were greater increases in the yoga + diet group following the 12‐weeks intervention compared to diet alone. In a study by Littman et al.^27^ breast cancer survivors with overweight or obesity were randomized to a 24‐weeks Hatha‐based yoga intervention (five session/week in‐person and at‐home) or a no‐intervention control group. MET‐hours/week of total PA and non‐yoga PA appeared to increase in the yoga arm and decreased slightly in the control arm; no *p*‐values were reported as this was a pilot study. In the study by Jakicic et al.^33^ comparing two yoga interventions, both groups increased PA, with and without adjustment for flights of stairs; the restorative yoga group had greater increases in several parameters of PA. Mama et al.^35^ observed higher self‐reported PA at follow‐up in the yoga intervention, although no *p*‐values were reported. There was also no change in minutes spent in moderate‐to‐vigorous PA in either group as measured by accelerometer. In the single‐arm study by Braun et al.^31^ increases in PA scores from the Health‐Promoting Lifestyle Profile II Questionnaire were observed directly following the 5‐days intervention (pre: 2.19 ± 0.73, post: 2.61 ± 0.77, *p* < 0.001). However, this increase included yoga completed in a residential setting and was not maintained at the 3‐months follow‐up.^31^ One investigation noted higher resting metabolic rate after the yoga intervention that was not observed in the control condition.^34^

Two RCTs explicitly instructed participants not to change habitual levels of PA. Despite instructions *not* to raise PA, Siu et al.^29^ observed increases in MET‐minutes/week from baseline to post‐intervention in both the yoga and control groups. However, these changes were not significantly different between groups, and *p*‐values were not reported for within‐group changes. In the pilot study by Cohen et al.^26^ no differences between groups in PA change were observed. However, results suggest that both changes in PA favored the yoga group in both PA hours/week and METs/week, though no *p*‐values were reported for within‐group changes.^26^

### 3.4 Bias

Most studies were likely to have a low (*n* = 3, 30%) or moderate risk of bias (*n* = 6, 60%); 1 (10%) had a high risk of bias (Table 2). In studies with pre‐post designs, the primary sources of bias were from not reporting or not measuring the following: whether all eligible participants were included, details of loss to follow‐up, statistical analyses, and measuring outcomes more than once during and after study periods. For RCTs, the main concerns were not reporting if the study team were blinded to the participants’ group assignments, adherence, and avoidance of other similar interventions. Drop‐out rates >20% and lack of intention‐to‐treat analyses were also common contributors to high risk of bias in RCTs.

**TABLE 2:** Risk of bias of included studies

Study	Question														Risk of bias
	1	2	3	4	5	6	7	8	9	10	11	12	13	14	
Pre‐post design															
Braun et al.^31^	Y	Y	N	NR	NR	N	N	CD	N	CD	N	NA	–	–	High
Telles et al.^32^	Y	Y	Y	NR	Y	Y	Y	Y	NR	N	N	NA	–	–	Moderate
Controlled interventions															
Cohen et al.^26^	Y	Y	Y	NA	NR	CD	Y	Y	Y	Y	Y	NA	Y	N	Low
Jakicic et al.^33^	Y	Y	Y	NA	NR	CD	Y	Y	N	NR	Y	NA	Y	Y	Moderate
Littman et al.^27^	Y	Y	CD	NA	NR	Y	Y	Y	Y	NR	Y	NA	Y	N	Low
Mama et al.^35^	Y	NR	CD	NA	NR	Y	Y	N	N	NR	Y	NA	Y	N	Moderate
Ruby et al.^28^	Y	CD	NR	NA	NR	CD	N	N	NR	NR	Y	N	Y	Y	Moderate
Siu et al.^29^	Y	Y	Y	NA	NR	Y	N	Y	NR	NR	Y	Y	Y	N	Moderate
Yadav et al.^30^	Y	Y	Y	NA	Y	Y	N	Y	NR	NR	Y	Y	Y	Y	Low
Yazdanparast et al.^34^	Y	Y	Y	NA	NR	Y	Y	Y	NR	NR	Y	Y	Y	N	Moderate

*Notes*: Questions are from the National Institutes of Health (NIH) Quality Assessment Tool for Before‐After (Pre‐Post) Studies with No Control Group and the NIH Quality Assessment of Controlled Intervention Studies (listed below). Each cell represents an assessment of each study, based on review and consensus from two reviewers (A.C. and S.P) as follows: Y, yes; N, no; CD, cannot determine; NA, not applicable; NR, not reported.

Pre‐post:1. Was the research question or objective clearly stated?2. Were the eligibility/selection criteria for the study population prespecified and clearly described?3. Were the participants in the study representative of those who would be eligible for the test/service/intervention in the general or clinical population of interest?4. Were all eligible participants that met the prespecified entry criteria enrolled?5. Was the sample size sufficiently large to provide confidence in the findings?6. Was the test/service/intervention clearly described and delivered consistently across the study population?7. Were the outcome measures prespecified, clearly defined, valid, reliable, and assessed consistently across all study participants?8. Were the people assessing the outcomes blinded to the participants' exposures/interventions?9. Was the loss to follow‐up after baseline 20% or less? Were those lost to follow‐up accounted for in the analysis?10. Did the statistical methods examine changes in outcome measures from before to after the intervention? Were statistical tests done that provided *p*‐values for the pre‐to‐post changes?11. Were outcome measures of interest taken multiple times before the intervention and multiple times after the intervention (i.e., did they use an interrupted time‐series design)?12. If the intervention was conducted at a group level (e.g., a whole hospital, a community, etc.) did the statistical analysis take into account the use of individual‐level data to determine effects at the group level?

Controlled Trials: 1. Was the study described as randomized, a randomized trial, a randomized clinical trial, or an RCT?2. Was the method of randomization adequate (i.e., use of randomly generated assignment)?3. Was the treatment allocation concealed (so that assignments could not be predicted)?4. Were study participants and providers blinded to treatment group assignment?5. Were the people assessing the outcomes blinded to the participants’ group assignments?6. Were the groups similar at baseline on important characteristics that could affect outcomes (e.g., demographics, risk factors, and co‐morbid conditions)?7. Was the overall drop‐out rate from the study at endpoint 20% or lower of the number allocated to treatment?8. Was the differential drop‐out rate (between treatment groups) at endpoint 15 percentage points or lower?9. Was there high adherence to the intervention protocols for each treatment group?10. Were other interventions avoided or similar in the groups (e.g., similar background treatments)?11. Were outcomes assessed using valid and reliable measures, implemented consistently across all study participants?12. Did the authors report that the sample size was sufficiently large to be able to detect a difference in the main outcome between groups with at least 80% power?13. Were outcomes reported or subgroups analyzed prespecified (i.e., identified before analyses were conducted)?14. Were all randomized participants analyzed in the group to which they were originally assigned, that is, did they use an intention‐to‐treat analysis?

## 4 DISCUSSION

Theoretically, yoga may influence several key determinants of health behavior change that support weight loss in people with obesity.^15^, ^16^, ^17^, ^18^, ^19^ Whether yoga‐induced weight loss occurs through decreased EI, increased PA, or both is unclear. As such, this systematic review examined the effect of yoga on EI and PA in adults with overweight and obesity. Our results suggest that the addition of yoga to a weight loss program may help reduce EI and improve several aspects of dietary intake,^30^, ^32^ but there is insufficient evidence to suggest that stand‐alone yoga interventions independently alter EI. Limited evidence suggests that self‐reported PA increases in response to a yoga intervention,^30^ though there is currently not consistent evidence that yoga is associated with increased non‐yoga PA. Importantly, in the only large RCT included in the systematic review where participants were *not* instructed keep habitual diet and PA patterns the same, the yoga + diet intervention led to greater decreases in EI and increases in PA than the diet intervention alone.^30^ Thus, while the overall body of literature is insufficient to definitively describe the effects of yoga on EI and PA among individuals with overweight and obesity, there is promising preliminary evidence that yoga can lead to improvements in both diet and PA, particularly when added to a lifestyle intervention. More large‐scale, rigorously designed studies are needed that are adequately powered to test for between group differences in changes in diet and PA.

There was also wide variability across studies included in this review in the different styles and specific yoga practices that comprised the yoga interventions that make comparing data across studies difficult. To date, there is no consensus on what styles of yoga should be included in interventions, which impedes rigorous scientific study of yoga and its potential health benefits. In the context of obesity or related chronic disease treatment, the intensity of the yoga practices, and the purpose of their inclusion (i.e., as a form of exercise vs. reducing appetite, pain, stress, or mitigating weight stigma) should be much more clearly considered, measured, and detailed in future research. Importantly, studies conducted in India and China placed a much greater emphasis on describing the specific yoga practices included in each intervention, and used practices more consistent with authentic yoga methods described in classic Sanskrit texts.^37^, ^38^ In contrast, studies in the United States included fewer details, used more recent interpretations of yoga (i.e., Kripalu^31^ or restorative^26^, ^33^), and incorporated other forms of exercise in the interventions (i.e., Pilates^28^). These interpretations emphasize the body‐based postures as a form of exercise and reflect the ways yoga has been commercially adopted, primarily as a form of exercise, in the West. However, other key elements of an authentic yoga practice, such as *pranayama* breathing and meditation techniques warrant inclusion in yoga intervention research in Western cultures as is common practice in the East. Reducing (or restricting) EI generally produces greater weight loss than exercise‐only interventions^39^ and is therefore critical for successful weight management. Cross sectional evidence suggests that yoga supports dietary intake practices and psychological changes that would support weight management including more servings of fruits and vegetables, fewer servings of sugar‐sweetened beverage and snack foods, less frequent fast‐food consumption, increased motivation to make healthier food choices, more mindful eating, and improved management of emotional eating and stress.^18^ However, this systematic review revealed that there is insufficient evidence to suggest that yoga interventions reduce EI in adults with overweight or obesity. While there appears to be modest within‐group changes,^26^, ^32^, ^33^ or positive alterations directly after a short‐term intervention,^31^ these results are not supported by group by time interactions (suggesting yoga does not alter dietary intake more or less than control conditions) or are not apparent after appropriate statistical adjustments. However, dietary intake was positively altered to a greater extent than a diet‐only only control group in one of the largest studies included in this review.^30^ Notably, all dietary data was ascertained using participant self‐reports, which are often inaccurate in people with obesity^40^, ^41^ and may have obscured legitimate alterations in dietary intake. The effects of a yoga intervention on objective EI and the determinants of such in adults with obesity therefore remain largely unknown.

There are several unique aspects of yoga that suggest yoga positively influences factors upstream could enhance EI‐related sustained behavior changes and support maintenance of weight loss. Improvements in subjective ratings of appetite occur following 10 minutes of slowed breathing (6 bpm vs. 9 bpm).^42^ Consistent slowed breathing exercises performed as part of a traditional yoga practice could subdue hunger, making reductions in EI easier to sustain over the long‐term. Yoga may also enhance self‐efficacy in regulating diet and PA^17^, ^43^ and reduce the frequency of binge eating,^44^ which would support lasting weight loss. In a qualitative study of individuals who lost weight through yoga, all respondents with overweight prior to weight loss described the process as involving a mindset shift to healthy eating, as well as improved mood, and emotional stability.^19^ Nearly all (90%) described it as a “different weight loss experience,” and many described that yoga led to more mindful eating, changes in food choices, and less emotional and/or stress eating.^19^ In summary, preliminary evidence suggests that yoga may improve key psychological and physiological characteristics that are fundamental for improved dietary intake. However, as highlighted by this systematic review, no study to date has assessed the direct impact of a yoga intervention on dietary intake or specific aspects of appetite in adults with overweight or obesity using rigorous measures.

PA is also important for weight loss and especially weight loss maintenance, as individuals who successfully maintain weight loss regularly engage in high levels of PA.^45^, ^46^ This is also reflected in current PA guidelines, which recommend 300 minutes/week of moderate intensity PA for weight management.^47^ In this review, all six studies that allowed participants to change PA,^27^, ^30^, ^31^, ^33^, ^34^, ^35^ and one where participants were instructed not to change PA,^29^ reported increases in self‐reported PA or resting metabolic rate after the intervention. However, only three reported *p*‐values for these changes,^30^, ^33^, ^34^ and one intervention was a 5‐days retreat in a residential setting and the observed increase in PA was not maintained among those completing a 3‐months follow‐up.^31^ Furthermore, objectively measured PA via accelerometers did not increase in one study, although there appeared to be increased self‐reported PA (*p*‐values not included).^35^ These results cautiously support the notion that PA may increase during a yoga intervention. However, it is important to note that many forms of yoga elicit low levels of EE^48^; it is therefore important to understand changes in non‐yoga PA and resting metabolic rate as this may have a more substantial impact on long‐term changes in TDEE. In other words, it may not be the EE associated with the yoga session that would substantially increase TDEE, but rather higher levels of EE from other components of TDEE. However, even a small increase in non‐yoga PA is promising, as it indicates that participants did not compensate for increases in yoga PA with reductions in other PA. Compensation can occur with moderate‐ and high‐intensity exercise and hinder weight loss.^49^, ^50^, ^51^ Although speculative, yoga may be an exercise modality, that is, *not* associated with compensation. This may be related to yoga eliciting a relatively lower EE compared to other, higher intensity exercise modalities.^48^ A previous study in older adults without obesity that demonstrated that a 6‐months yoga intervention did not result in altered total, resting, or PA EE.^52^ This review also included one study that reported increased resting metabolic rate (∼100 kcal/day), although this was not assessed in conjunction with body composition to contextualize results and was measured with a portable indirect calorimeter, which may have poor accuracy^53^, ^54^ Further research investigating objectively measured non‐yoga PA, resting metabolic rate, and TDEE during a yoga intervention in people with obesity is warranted.

This systematic review highlights a paucity of research that has characterized the effects of yoga interventions on PA in adults with overweight or obesity. However, other related research in populations that are not exclusively overweight or obese suggests that yoga practice is expected to improve PA levels though mechanisms that are not related to the assumed increased EE from yoga sessions. Bernstein et al.^15^ suggested that yoga can improve PA participation in adults with obesity through reductions in back and joint pain. In a 10‐weeks yoga intervention to prevent weight gain among Puerto Rican college students, self‐reported walking increased in the yoga arm, but decreased in the sedentary control arm.^55^ In a cross‐sectional epidemiological study of 15,550 adults age 50 to 76 years old, individuals with >4 years of yoga practice reported more than two‐fold higher PA than individuals who did not engage in yoga.^56^ In a non‐obese sample, yoga practice increased positive affect, physical function, and self‐esteem during a 20‐weeks behavioral weight loss intervention with mind‐body techniques (i.e., meditation, imagery).^17^ Finally, among yoga practitioners in a nationally representative survey, nearly two‐thirds said that doing yoga motivated them to eat healthier (63%) and exercise more regularly (63%).^57^ Thus, there is strong rationale for future studies to test the extent to which yoga leads to changes in total and non‐yoga PA levels in adults with obesity using objective measures to address remaining gaps in the literature.

This review is the first to describe changes in EI and PA in response to yoga in people with obesity and uncovered several limitations that should be considered when interpreting the literature. The widespread reliance on self‐reported measures of dietary intake and PA is the most important limitation of the current evidence. These measures have poor accuracy in identifying actual EI and PA,^40^, ^41^, ^58^ and therefore impede current understanding of how engaging in yoga practice may influence other aspects of health behavior. Error is especially pronounced with abbreviated methods of dietary intake and PA such as 24‐hour diet recalls and PA questionnaires, as they do not capture variability in behavior. Furthermore, the use of various self‐report measures and different metrics from the same measure precludes the ability to compare findings across studies. Future research using more rigorous techniques for assessment of EI, appetite, PA, and total EE (e.g., doubly labeled water, accelerometers, meal studies, and food photography) is warranted and essential. Current interpretation is also limited by the guidelines on health behaviors individuals received in several interventions. Instructions to avoid changes in EI and PA obscures any independent changes and likely diminishes the true effect of yoga on EI and PA. There was also a wide variability in the type of yoga in each intervention, with some providing more details about the intervention than others. The array of different methodologies regarding participant instructions and yoga interventions also hampers a rigorous comparison of studies. A limitation of this particular review is that a meta‐analysis was not possible given the differences in study design and outcomes measures.

In conclusion, there is currently only limited evidence that yoga improves EI and PA parameters in adults with overweight or obesity. However, this is at least partially due to the small number of studies that have measured these outcomes in this population in response to a yoga intervention (most with small sample sizes), the wide variability in study designs, and lack of rigorous, objective measurements of these variables. The rationale for examining the effects of yoga on EI and PA during weight loss is compelling and suggests that yoga holds promise as a strategy to support weight loss and weight loss maintenance in people with obesity. Whether yoga may elicit changes in energy balance as a stand‐alone intervention or as part of a comprehensive lifestyle modification interventions is unknown. Rigorously designed research with objective measures of energy balance is warranted to measure the effects of yoga on EI and PA to understand how yoga leads to weight loss in this population in order to reduce the burden of obesity.

## CONFLICTS OF INTEREST

The authors declare no conflicts of interest.

## AUTHOR CONTRIBUTIONS

Ann E. Caldwell, Sarah A. Purcell, and Victoria A. Catenacci conceptualized the review. Ann E. Caldwell and Sarah A. Purcell developed and implemented the search strategy. BAG and HS conducted the title and abstract screening. Ann E. Caldwell and Sarah A. Purcell reviewed the selected abstracts and full texts, extracted and analyzed the data, reviewed the risk of bias, and wrote the original draft of the manuscript. All authors provided critical feedback on the manuscript.

## Supporting information

Supporting Information S1
