# Supplementary material for: The impact of yoga on components of energy balance in adults with overweight or obesity: A systematic review
Source: Obes Sci Pract. 2021 Aug 19;8(2):219–32. doi: 10.1002/osp4.552 (PMC8976548; doi:10.1002/osp4.552)
Supplement: Supplementary file 1 — Supporting Information S1 [file OSP4-8-219-s001.pdf]

Appendix 1

Search terms and results
4/10/2020

**PubMed**

((((("yoga"[MeSH Terms] OR "yoga"[All Fields]) OR "yogic"[All Fields]) OR ("asana"[All Fields] OR "asanas"[All Fields])) OR ("pranayama"[All Fields] OR "pranayamas"[All Fields])) AND (((((((((((((((((((((("energy intake"[All Fields] OR "food intake"[All Fields]) OR "energy balance"[All Fields]) OR "energy consumption"[All Fields]) OR "diet*"[All Fields]) OR ((((("appetite"[MeSH Terms] OR "appetite"[All Fields]) OR "appetites"[All Fields]) OR "appetitive"[All Fields]) OR "appetitively"[All Fields]) OR "appetitiveness"[All Fields])) OR (("hunger"[MeSH Terms] OR "hunger"[All Fields]) OR "hungers"[All Fields])) OR ((("satiation"[MeSH Terms] OR "satiation"[All Fields]) OR "satiety"[All Fields]) OR "satieties"[All Fields])) OR ((((("satiate"[All Fields] OR "satiated"[All Fields]) OR "satiating"[All Fields]) OR "satiation"[MeSH Terms]) OR "satiation"[All Fields]) OR "satiations"[All Fields])) OR "fullness"[All Fields]) OR ("eating"[MeSH Terms] OR "eating"[All Fields])) OR "restrain*"[All Fields]) OR ((((("disinhibit"[All Fields] OR "disinhibited"[All Fields]) OR "disinhibiting"[All Fields]) OR "disinhibition"[All Fields]) OR "disinhibitions"[All Fields]) OR "disinhibits"[All Fields])) OR ("eating"[MeSH Terms] OR "eating"[All Fields])) OR "energy metabolism"[All Fields]) OR "metabolic rate"[All Fields]) OR "energy expenditure"[All Fields]) OR "physical activity"[All Fields]) OR "exercise"[All Fields]) OR "sedentary*"[All Fields]) OR "non-exercise activity"[All Fields]) OR "non-exercise physical activity"[All Fields]) OR "NEAT"[All Fields]) OR "NEPA"[All Fields])) AND (((((((((("obes*"[All Fields] OR (((("overweight"[MeSH Terms] OR "overweight"[All Fields]) OR "overweighted"[All Fields]) OR "overweightness"[All Fields]) OR "overweights"[All Fields])) OR "weight loss"[All Fields]) OR "weight management"[All Fields]) OR "BMI"[All Fields]) OR "body mass index"[All Fields]) OR "body composition"[All Fields]) OR "lean body mass"[All Fields]) OR "lean tissue"[All Fields]) OR "body fat"[All Fields]) OR "fat mass"[All Fields]) OR "waist circumference"[All Fields])

Translations

Yoga: "yoga"[MeSH Terms] OR "yoga"[All Fields]

asana: "asana"[All Fields] OR "asanas"[All Fields]

pranayama: "pranayama"[All Fields] OR "pranayamas"[All Fields]

appetite: "appetite"[MeSH Terms] OR "appetite"[All Fields] OR "appetites"[All Fields] OR "appetitive"[All Fields] OR "appetitively"[All Fields] OR "appetitiveness"[All Fields]

hunger: "hunger"[MeSH Terms] OR "hunger"[All Fields] OR "hungers"[All Fields]

satiety: "satiation"[MeSH Terms] OR "satiation"[All Fields] OR "satiety"[All Fields] OR "satieties"[All Fields]

satiation: "satiate"[All Fields] OR "satiated"[All Fields] OR "satiating"[All Fields] OR "satiation"[MeSH Terms] OR "satiation"[All Fields] OR "satiations"[All Fields]

eating: "eating"[MeSH Terms] OR "eating"[All Fields]

disinhibition: "disinhibit"[All Fields] OR "disinhibited"[All Fields] OR "disinhibiting"[All Fields] OR "disinhibition"[All Fields] OR "disinhibitions"[All Fields] OR "disinhibits"[All Fields]

eating: "eating"[MeSH Terms] OR "eating"[All Fields]

overweight: "overweight"[MeSH Terms] OR "overweight"[All Fields] OR "overweighted"[All Fields] OR "overweightness"[All Fields] OR "overweights"[All Fields]

Web of Science

ALL FIELDS: (Yoga OR yogic OR asana OR pranayama) AND ALL FIELDS: ("energy intake" OR "food intake" OR "energy balance" OR "energy consumption" OR diet* OR appetite OR hunger OR satiety OR satiation OR fullness OR eating OR restrain* OR disinhibition OR eating OR "energy metabolism" OR "metabolic rate" OR "energy expenditure" OR "physical activity" or "exercise" or "sedentary*" OR "non-exercise activity" OR "non-exercise physical activity" OR NEAT OR NEPA) AND ALL FIELDS: (Obes* OR overweight OR "weight loss" OR "weight management" OR "BMI" or "body mass index" OR "body composition" OR "lean body mass" or "lean tissue" OR "body fat" or "fat mass" OR "waist circumference")

Timespan: All years. Indexes: SCI-EXPANDED, SSCI

Embase

(yoga OR yogic OR asana OR pranayama) AND ('energy intake' OR 'food intake' OR 'energy balance' OR 'energy consumption' OR diet* OR appetite OR hunger OR satiety OR satiation OR fullness OR restrain* OR disinhibition OR eating OR 'energy metabolism' OR 'metabolic rate' OR 'energy expenditure' OR 'physical activity' OR exercise OR sedentary* OR 'non-exercise activity' OR 'non-exercise physical activity' OR neat OR nepa) AND (obes* OR overweight OR 'weight loss' OR 'weight management' OR 'bmi' OR 'body mass index' OR 'body composition' OR 'lean body mass' OR 'lean tissue' OR 'body fat' OR 'fat mass' OR 'waist circumference')

#5 AND (2015:py OR 2016:py OR 2017:py OR 2018:py OR 2019:py OR 2020:py)

PsychINFO

1. (Yoga or yogic or asana or pranayama).mp. [mp=title, abstract, heading word, table of contents, key concepts, original title, tests & measures, mesh]
2. (energy intake or food intake or energy balance or energy consumption or diet* or appetite or hunger or satiety or satiation or fullness or eating or restrain* or disinhibition or eating or energy metabolism or metabolic rate or energy expenditure or physical activity or exercise or sedentary* or non-exercise activity or non-exercise physical activity or NEAT or NEPA).mp. [mp=title, abstract, heading word, table of contents, key concepts, original title, tests & measures, mesh]
3. (Obes* or overweight or weight loss or weight management or BMI or body mass index or body composition or lean body mass or lean tissue or body fat or fat mass or waist circumference).mp. [mp=title, abstract, heading word, table of contents, key concepts, original title, tests & measures, mesh]

4. 1 and 2 and 3
